# Insights into secular trends of respiratory tuberculosis: The 20th century Maltese experience

**DOI:** 10.1371/journal.pone.0183296

**Published:** 2017-08-17

**Authors:** Lianne Tripp, Larry A. Sawchuk

**Affiliations:** Department of Anthropology, University of Toronto at Scarborough, Toronto, Ontario, Canada; Institut de Genetique et Microbiologie, FRANCE

## Abstract

Over half a century ago, McKeown and colleagues proposed that economics was a major contributor to the decline of infectious diseases, including respiratory tuberculosis, during the 19^th^ and 20^th^ centuries. Since then, there is no consensus among researchers as to the factors responsible for the mortality decline. Using the case study of the islands of Malta and Gozo, we examine the relationship of economics, in particular, the cost of living (Fisher index) and its relationship to the secular trends of tuberculosis mortality. Notwithstanding the criticism that has been directed at McKeown, we present results that improvement in economics is the most parsimonious explanation for the decline of tuberculosis mortality. We reaffirmed that the reproductively aged individuals were most at risk of dying of tuberculosis, seeing that 70 to 90% of all deaths due to tuberculosis occurred between the ages of 15 and 45. There was a clear sex differential in deaths in that, prior to 1930, rates in females were generally higher than males. During times of extreme hardship, the sex differential was exacerbated. Over the course of World War I, the sex gap in tuberculosis rates increased until peaking in 1918 when there was also the influenza pandemic. The heightened differential was most likely a result of gendered roles as opposed to biological differences since female tuberculosis rates again surpassed male rates in 1945 during World War II. Respiratory tuberculosis in both urban and rural settlements (in Malta proper) was significantly influenced by the Fisher index, which explains approximately 61% of the variation in TB death rates (R = 0.78; p<0.0001). In Gozo, there was no significant impact on respiratory tuberculosis (R = 0.23; p = 0.25), most likely a consequence of the island’s isolation, a self-sufficient economy and limited exposure to tuberculosis.

## Introduction

As a re-emerging infectious disease of considerable importance, respiratory tuberculosis is one of the top ten causes of death worldwide [1] and until recently was the single main cause of death worldwide in young adults [2–5]. The history of tuberculosis in humans, contrary to popular belief, predates the Neolithic period and domestication [6, 7]. By at least 50 A.D., tuberculosis decimated populations in Europe [8], killing, for the most part, those in their reproductive prime. Beginning around eighteen hundred, however, there was a gradual and progressive decline in respiratory tuberculosis death rates [9–11]. As the secular trend in decreasing rates preceded knowledge of the etiology of tuberculosis and use of effective anti-tuberculosis drugs, reasons for the decline are the subject of considerable debate and remain elusive. Leading explanations include a number of factors. Better living conditions resulted in an improved diet and, possibly, a favourable trend in the relationship between the bacilli and the human host [10, 12]. Government initiatives aimed at public health measures were able to reduce "urban congestion" [13]. The average number of “effectively contacted” by an infectious case decreased for the following reasons: the decline in household crowding; improved ventilation in buildings; reduction in the proportion of the elderly, who are sources of tuberculous infection, residing at home; and the segregation of the infected to workhouses or sanatoria [11] and possibly, due in part to the action of natural selection [14, 15].

The global decline in tuberculosis deaths was interrupted twice—during World War I and to a lesser extent during World War II—and then resumed in post-war years [16]. Undoubtedly, wartime conditions played a huge role in promoting infections of tuberculosis by hastening the conversion of latent tuberculosis to active tuberculosis cases [9]. During World War I, rates of respiratory tuberculosis reached unprecedented levels when even nonbelligerent countries experienced strikingly high tuberculosis death rates. In some of the countries at war such as those in the entire British Empire, more people died of tuberculosis in the single year of 1917 (over 1 million) than soldiers on the battlefield during the course of the four plus years of war [17]. Nevertheless, war conditions alone could not have accounted for the sheer magnitude of tuberculosis death rates. According to one theory, a novel strain of *M*. *tuberculosis*, the Beijing lineage, resurfaced during World War I—a strain with a quicker transition from infection to disease, increased virulence, and transmissibility [18]—and would have been responsible for the increased mortality. Following the war, there was a sudden return to the declining trend of pre-war tuberculosis rates suggesting the strain alone could not have been the main contributor to the elevated rates: the bacilli would need to have disappeared abruptly from the population after the end of the war. Additional theories about contributing factors to the increase of rates during the First World War are as follows: overcrowding, which would have been more noticeable in war countries where women, children, and refugees were displaced from war torn areas; increase in malnutrition, although there is evidence allied countries did not experience food shortages to the same extent as German occupied nations; shortage of medical care [9, 16, 19]; and the selective effect of influenza on the tuberculous during the 1918 pandemic [20]. The influenza and tuberculosis relationship can account only for the increase in 1918/19 and not in the preceding war years. Together, the events of World War I and pandemic influenza constitute a singular event that triggered population shock and, in turn, its effect on tuberculosis mortality was unprecedented. In England and Wales, for example, tuberculosis deaths in 1913 was 135 per 100 000, rose to 162 in 1917, and then fell to 113 per 100 000 in 1920 [17]. We contend that such rare and fortuitous events can be a useful tool to gain insight into the relative importance of specific factors that normally lie hidden in diseases of complex etiology.

In this paper, we explore reasons for the decline of tuberculosis death rates in the civilian population of Malta during the early 20^th^ century. We also address the question of why there were deviations from the normative trend in 1917 and 1918 when rates exceeded background mortality. We examine overall and regional trends in tuberculosis by sex, age, and sex-age specific rates as well as changes over time and during the crisis period of World War I.

### The study population: Background on Malta

The Malta archipelago consists of three major islands: Malta, Gozo, and Comino. Because Comino is sparsely populated, our study does not include this island. Historically, on the larger island of Malta, there existed significant regional differences in terms of landscape: an urban and rural dichotomy. With an area of approximately sixty-seven square kilometres and lying five kilometres away from the main island, Gozo is about one quarter the size of Malta. Malta represents an ideal location to study secular trends in tuberculosis as it minimizes a number of confounding factors responsible for elevated rates of air-borne infections or obscuring real trends in tuberculosis mortality. First, prior to World War II, Malta was a geographically isolated population with little immigration. Consequently, the effects of immigration from high prevalence countries which could have altered the rates of tuberculosis over time can be viewed as minimal [21]. Second, there was little large scale industry [22, 23] to expose major segments of the population to airborne pollutions. Industrial emissions could have compromised the overall health of the population and exacerbated infections such as respiratory tuberculosis. Finally, the potential impact of exposure to *M*. *bovis* prior to the pasteurization of milk in 1950 and its subsequent impact on tuberculosis and reactivation [24], can be considered negligible because Malta was completely reliant on goat's milk rather than cow’s milk prior to World War II (see [25]).

Aside from the features of cultural and religious homogeneity, there is an important element of diversity in occupation and economics that allows for a better understanding of changes in tuberculosis mortality. The island of Malta’s topography, with its marginal *Xaghri* (karstik land) and overall scarcity of productive land, meant that the Maltese were largely dependent on food imports for their nutritional needs [26]. There were, however, marked differences within the Maltese landscape for food self-sufficiency. Inhabitants of Gozo and rural Malta farmed their land, thus these locales were less reliant on imported food products. By examining tuberculosis in the three regional settings (Gozo, Malta suburban/urban, and Malta rural), we may gain a deeper understanding of nutrition and its role in tuberculosis deaths. In other words, the Maltese setting can be viewed as a continuum of populations residing in areas of high dependency to low dependency on food imports.

Gozo’s distinct cultural ethos and biological heritage is grounded in geographical isolation. Gozitans see themselves ‘apart’ from the Maltese for several reasons: they feel they have been marginalized by bureaucratic neglect, overlooked in regard to fundamental economic opportunities and denied adequate health and social infrastructure [27]. Because the island was so small and its people showed a long-standing preference for spatial endogamy, it comes as no surprise that, prior to World War II, there were scarcely any differences to be found between the life and customs in the town and in the villages [28].

Accordingly, we shall take advantage of conditions equivalent to a “natural experiment” where the impact of a moment of crisis associated with World War II and the 1918 influenza pandemic combined with an unusual population setting can be studied for its effect on respiratory tuberculosis mortality. These conditions include the following: a high impact stressor that targeted a significant portion of the population and very likely had a negative and discernible impact on health because of its known biological and environmental etiology; a stressor that occurred over a clearly defined period of time and one of sufficient temporal scope to yield measurable results. The second factor, the population, was broadly biologically and culturally homogenous and yet was diverse enough evaluating the importance of potential risk factors on vulnerable segments of the community.

## Methods and materials

To assess the overall health status of males and females in the Maltese islands, we used life table analysis. We employed life expectancy estimates at birth and at age twenty-five for the years 1911 to 1924. Life expectancy estimates at the age of twenty-five provides a proxy measure of health of the reproductively aged individuals, the age category most at risk of pulmonary tuberculosis, and they provide a more accurate indicator of adult health. Traditionally, the results from life tables have been used to assess the health of large populations. Recent studies, however, have based their studies of smaller populations on the life table methodology (see [29, 30]).

Using the Smith Survival Program (Version 9.2) [31], we applied the Chiang period approach [32] for estimating various life table parameters. The benefits of employing the Chiang method are these: (1) it produces the most conservative estimates for comparison between local areas; (2) it is easy to calculate (including statistical variance); (3) it allows for sensitivity analysis to perform on the major assumptions; and (4) it is widely used in research allowing for comparability of results to other populations [29].

To capture the nature of the change in tuberculosis mortality by age and sex, we constructed four discrete time periods: (a) 1911 to 1916; (b) 1917; (c) 1918; and (d) 1919 to 1924. Results from our life table analysis framed the probability of dying from respiratory tuberculosis under the prevailing mortality pattern.

Annual deaths numbers by sex and age for all causes, tuberculosis and influenza were drawn from the Health Reports for the Maltese islands (1911–1938) and from the Maltese Gazette for monthly causes of death by sex and age (1939–1952). Published under the auspices of the Medical Officer of Health, the reports also yielded information on health related matters about infants, maternity sanitation, housing, food quality, water as well as detailed accounts and observations of morbidity of notifiable diseases over the course of a year.

To reconstruct the population at risk for the population and its respective regions, we took advantage of published census reports from 1911, 1921, 1931, and 1948. Annual sex and age specific population at risk taken at four-year intervals beginning with one year olds was interpolated by using age-specific multipliers applied to the age and sex specific population sizes from the census of the closest year. The total population, we estimated, fell within a margin of less than 0.5% of the total population as cited in the annual Health Reports. Yearly birth rates, retrieved and compiled from the monthly Malta Gazette death records where live birth information was recorded, gave the approximate number of infants under one year of age. This approach assumed that stability in the proportions of individuals at risk for each age band over the decennial period. Using the aforementioned data sources, we compiled the overall as well as the sex and age specific (15 to 44 years) annual tuberculosis mortality rates for Malta from 1911 to 1949.

We recognize potential limitations in historical studies of tuberculosis. Since tuberculosis was often incorrectly identified as the cause of death [33], it is inherently possible that we overestimated our rates of tuberculosis mortality. At the same time, there was no sure method of confirming cases and causes of death even by World War I when portable x-rays were used, but not readily available, so that many were diagnosed without the confirmation of x-rays [9]. For this latter reason, some have argued that the improvement in diagnosis over time has been partly responsible for the decline in tuberculosis deaths [33].

Using census returns and their protocol for defining the residential settlement type, we divided Malta’s settlements into three distinct settings: (1) urban and suburban setting, (2) rural setting, and (3) Gozo. We used Malta’s administrative definition of the respective regions as specified in the Census returns of 1911 and 1921, and the Annual Health Reports.

Because of the varying degrees of dependency on the importation of foodstuff across the Maltese islands, we used food imports as a proxy measure of the cost of living, which can be examined for its effectiveness as an explanatory variable for the rates of respiratory tuberculosis mortality over time and within the three regions. Data on economic parameters used to construct the price and quantity index, the chained linked Fisher index, for 1910 to 1938 (years 1916 and 1920 were excluded from the study), was sourced from the importation of goods information found in the Malta Blue books. J. Falzon generously supplied the values for the Fisher index as presented in Falzon and Lanzon, 2011 [34]. We chose it for its superiority as a price and quantity index because it satisfies most of the desirable properties of an index (see the 10 properties as outlined in [34] and as originally presented by Diewert, 1987 [35]). The Fisher index is a measure of inflation based on house consumption and unit price values, and is the square root of the Laspeyres index multiplied by the Paasche index. We analyzed the relationship of economics to tuberculosis death rates (overall and regional) using least square regression and Pearson’s correlation analysis. Data on tuberculosis deaths, by sex and age, and population at risk are included in the supporting information (See S1 Dataset).

## Results

### Background health

**Fig 1:**
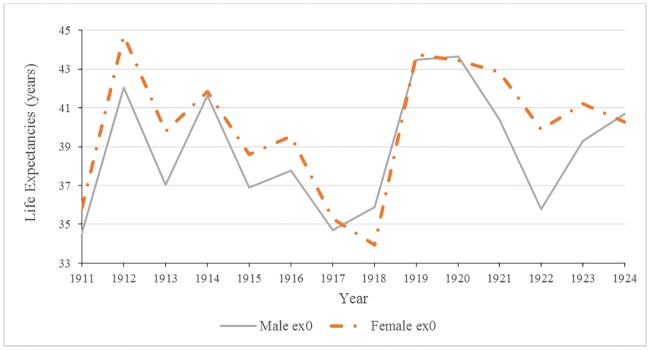
Male and female life expectancy at birth: Malta 1911 to 1924.

Fig 1 shows that during the thirteen year study period life expectancy at birth did not exceed 44 years, however life expectancy at birth was highly variable for one year to another as life at birth in Malta was tenuous. Diarrheal deaths accounted for high infant mortality rates, and harsh climatic conditions such as an unseasonably dry hot summer would have exacerbated infant deaths [36].

**Fig 2:**
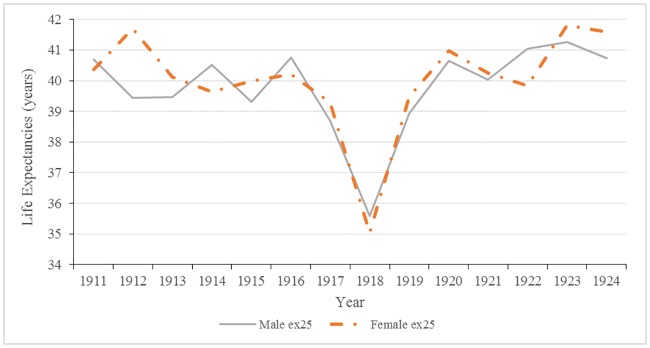
Male and female life expectancy at 25 years of age: Malta 1911 to 1924.

As shown in Fig 2, life expectancy from 20–44 years of age for both males and females remained relatively stable over the study period, with the exception of 1918. With a maximum life expectancy of 44 years, the total average lifespan was approximately 66 years.

**Fig 3:**
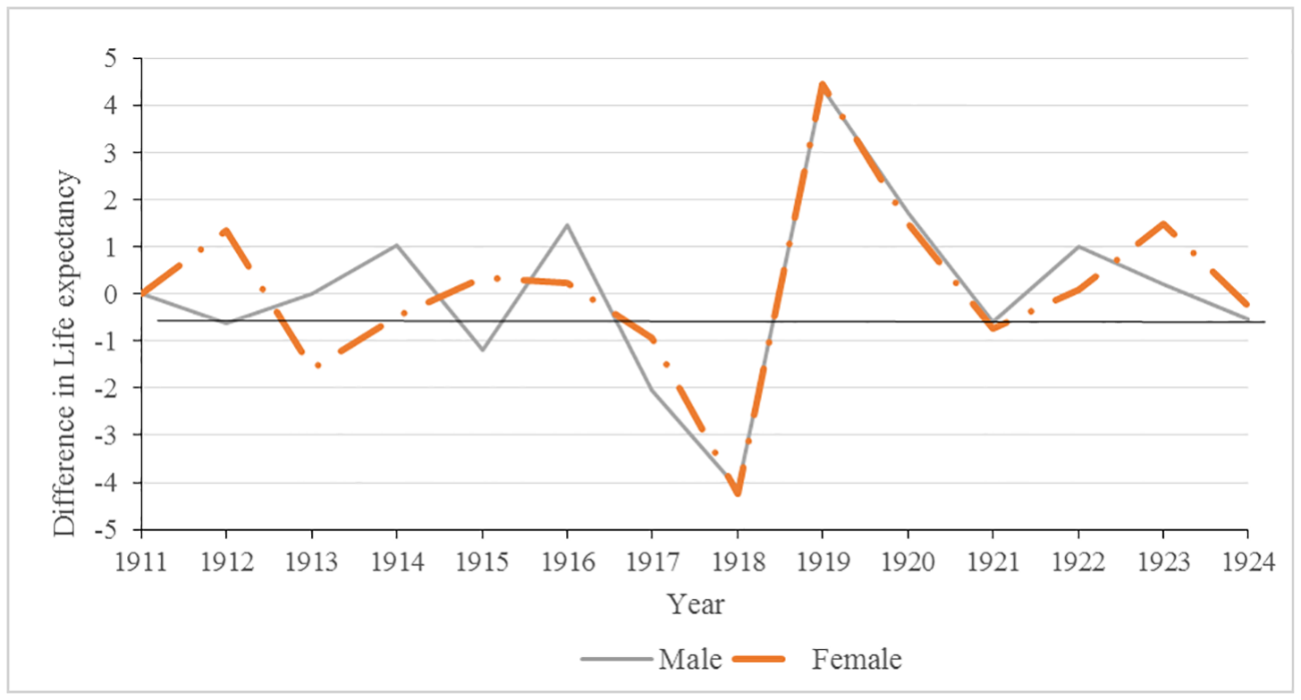
Male and female changes in annual life expectancies at 25 years of age: Malta 1911–1924.

Annual changes in life expectancies at 25 years are shown in Fig 3. Significant decreases in male life expectancies occurred in 1915, 1917, and 1918 (p-values <0.05). In females, the only significant drop in life expectancy at 25 years occurred in 1918; the impact of the 1918 influenza epidemic on the overall health of the population cannot be overlooked. During this year alone, life expectancy plummeted to an all-time low of 35.58 years for males and 35.04 years for females, a four year decrease from 1917. Following the war in 1919 and 1920, there were significant changes: increases in life expectancy, rebounding or, in some years, exceeding pre-war levels.

### Probability of dying from respiratory tuberculosis

**Fig 4:**
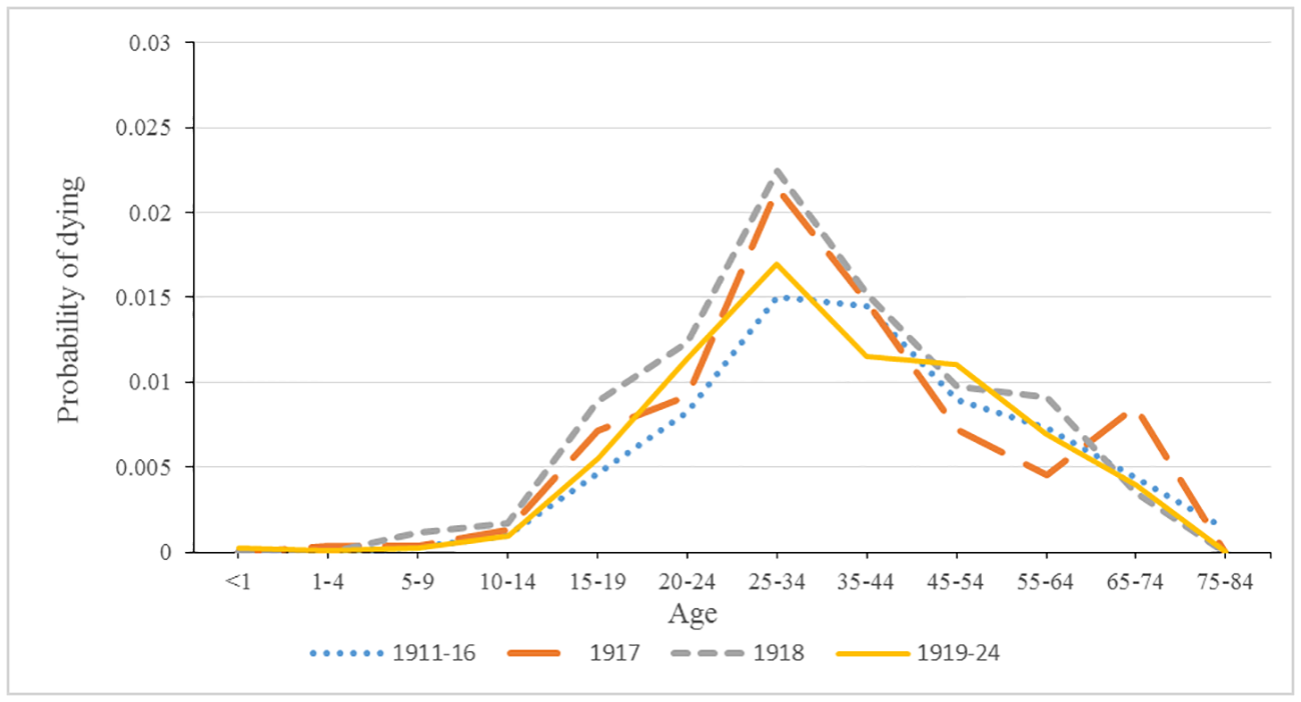
Male probability of dying from tuberculosis for four time periods.

**Fig 5:**
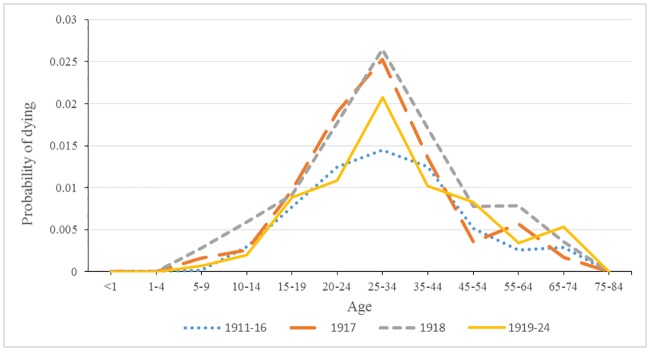
Female probability of dying from tuberculosis for four time periods.

Figs 4 and 5 show the distinctive pattern of the rise in tuberculosis mortality for both 1917 and 1918 relative to the period before and after the dramatic rise in tuberculosis mortality. For males, the increase was concentrated in the age bands 25 to 34 years and 35 to 44 years. It is also noteworthy that probability of dying from respiratory tuberculosis after 1918 was very similar to the pattern before 1917. The female pattern of tuberculosis mortality was different in 1917 and 1918 from that of males in terms of a broader base of heightened mortality, as well as a larger peak in mortality. Following 1918, respiratory tuberculosis mortality retained its distinctive peak at age band 25 to 34 and remained higher than the period preceding 1917.

### Respiratory tuberculosis rates over time

**Fig 6:**
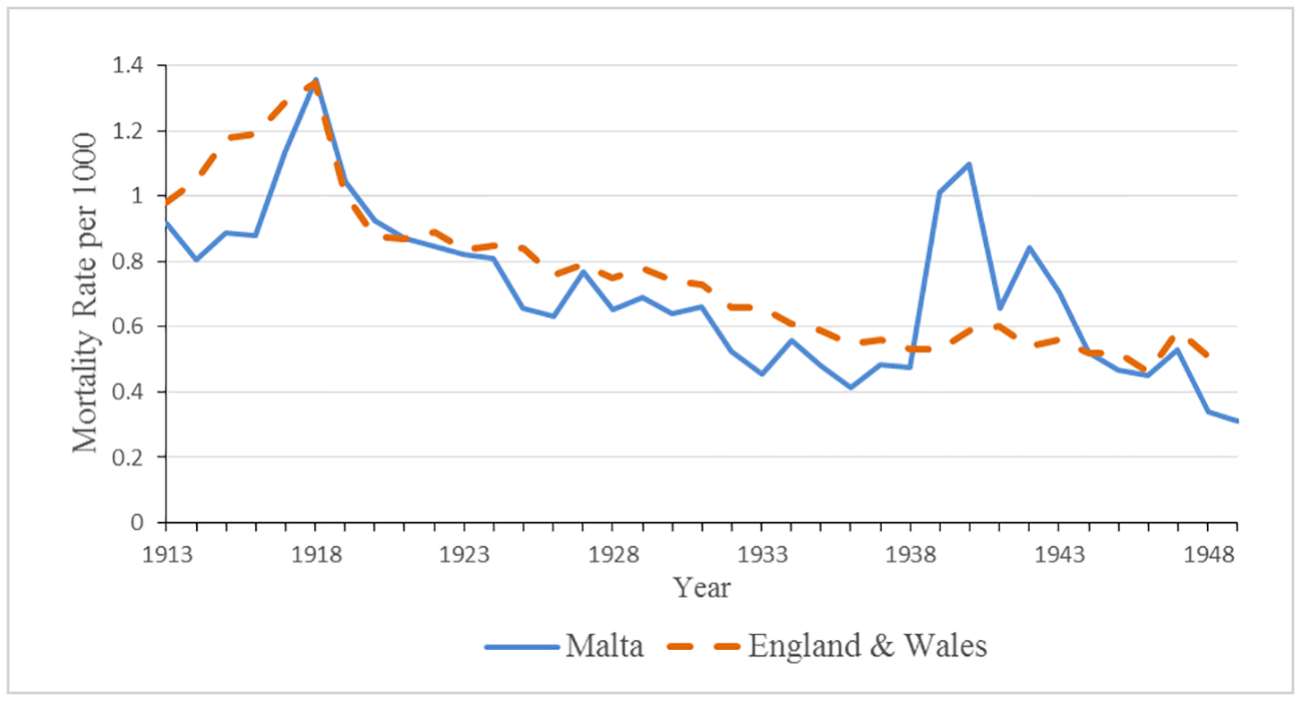
Overall tuberculosis mortality rate for Malta and England 1913–1949.

As is shown in Fig 6, the general trend for tuberculosis deaths rates from the early to mid-1900s in the Maltese islands was a gradual decline. During World Wars I and II, the secular trend was interrupted, and rates rose to exceptional levels, 1.36 deaths per 1000 individuals in 1918, and to a lesser magnitude of 0.84 deaths per 1000 individuals in 1942. Similar findings of exceptionally high mortality rates during the world wars have been observed elsewhere (see [9, 17, 37]). The markedly high tuberculosis rates during 1917 and 1918 are attributed by most scholars to the confluence of the war and the influenza epidemic. In comparison to England, overall tuberculosis death rates were lower but similar in pattern, with the exception of World War II when rates in Malta greatly surpassed England (see Fig 6).

**Fig 7:**
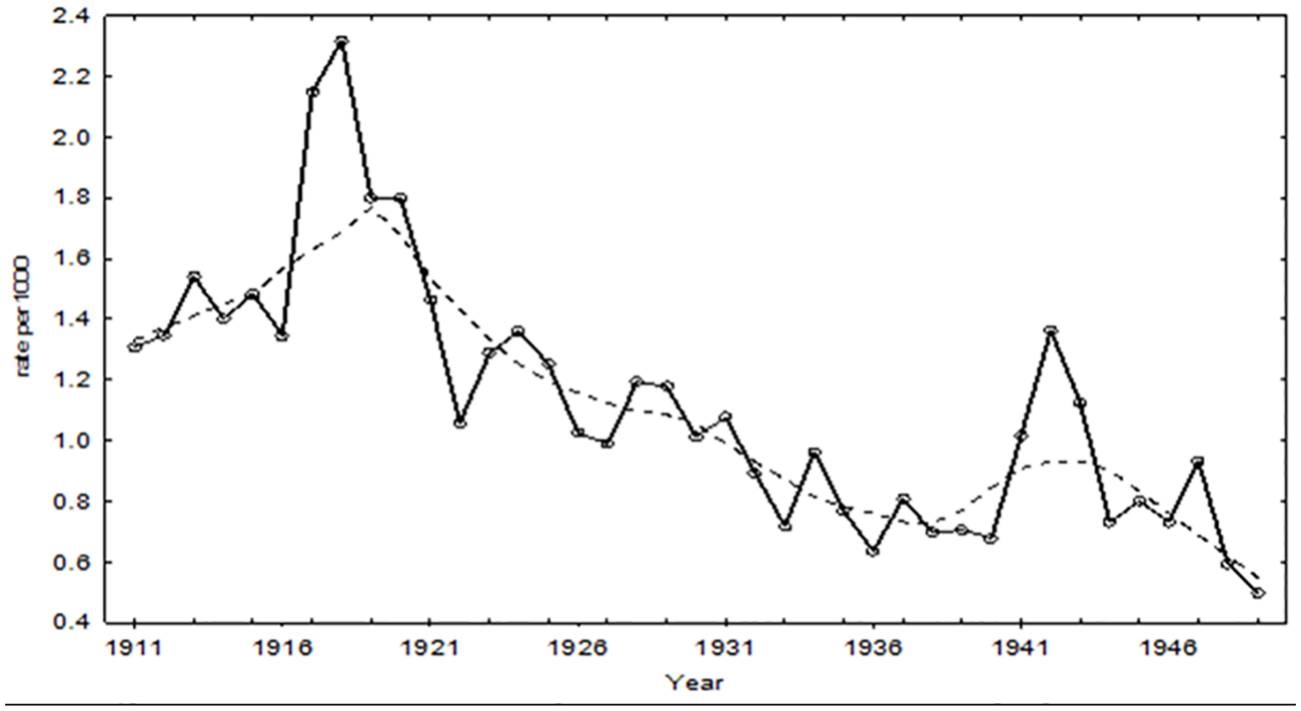
Tuberculosis mortality rate and lowess for reproductively aged individuals: Malta 1915–1949.

The mortality rates in the reproductively aged adults provide a more refined exploration of the chronological trend in tuberculosis mortality rates since, in any given year, 70 to 90% of all deaths due to tuberculosis occurred between the ages of 15 and 45 years of age. Fig 7 shows that the general trend of a reduction in tuberculosis rate remains the same, but the absolute rates during the war were more pronounced: 2.32 deaths per 1000 individuals and 1.36 per 1000 individuals in 1918 and 1942 respectively. There was a decline in rates beginning in 1920, reaching the nadir in 1922 and rebounding to almost pre-war levels in 1923.

**Fig 8:**
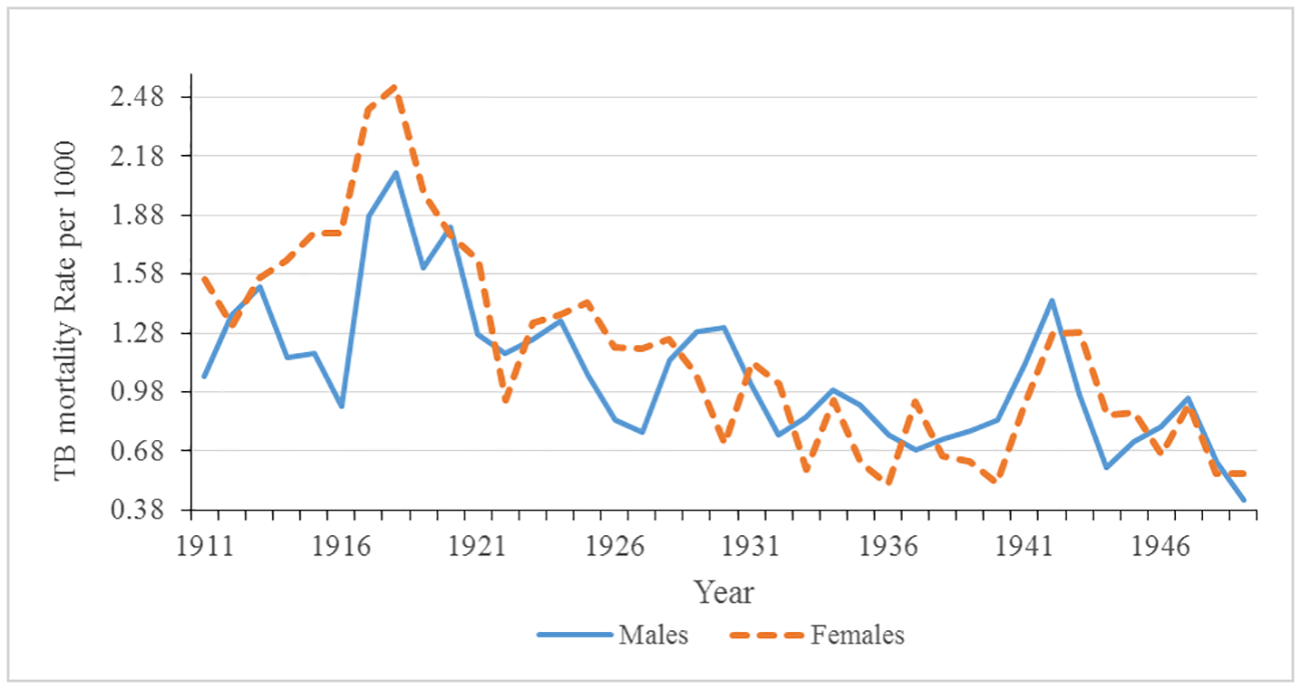
Male and female tuberculosis mortality rates: Malta 1911–1949.

**Fig 9:**
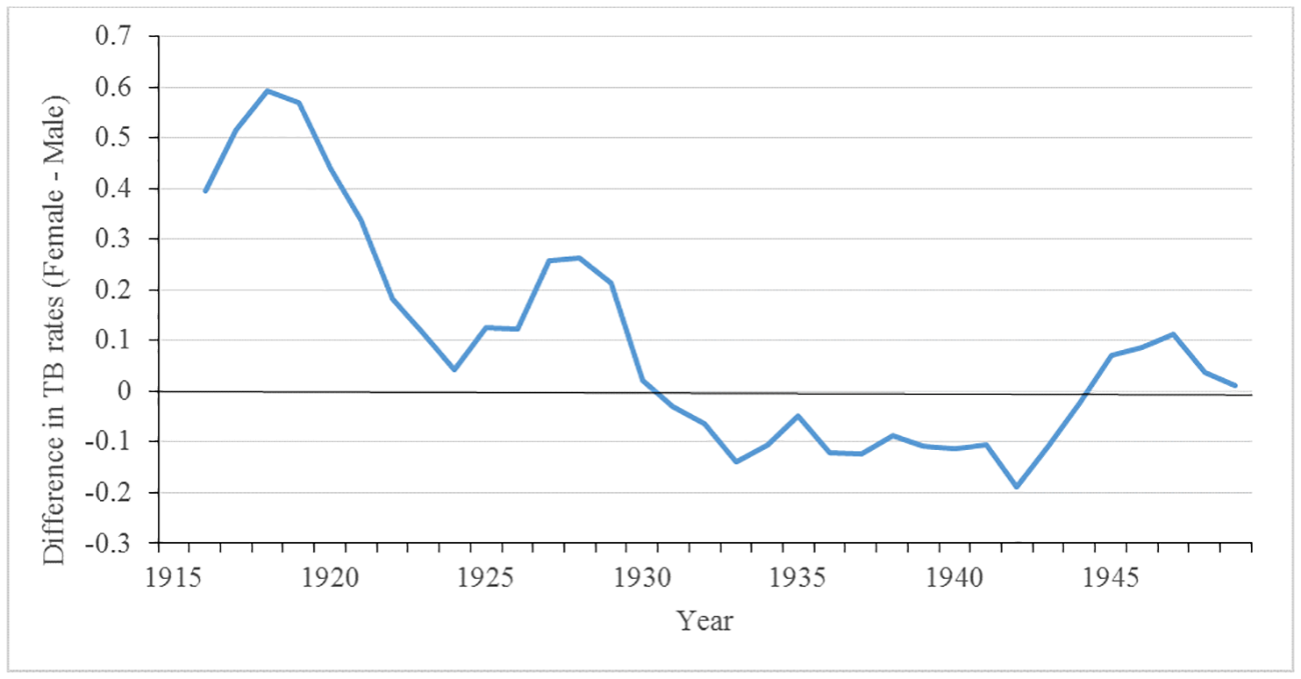
Absolute sex differences in tuberculosis mortality rates: 1911–1949*. *difference is = Female TB mortality rate- Male TB mortality rate.

Fig 8 shows that the tuberculosis death rates for males and females; rates in females were generally higher than male rates. We observed a trend in reproductively aged females similar to the overall tuberculosis death rates: there was spike of tuberculosis during the war that peaked in 1918, followed by a rapid decline thereafter, with an increase close to pre-war levels. On the other hand, the trend in male tuberculosis rates during the war and post-war period differed from that of the females. Male tuberculosis rates dipped only slightly in 1919 when the third wave of the influenza epidemic was occurring, and then returned to near pre-war levels in 1920. It follows that reproductively aged females rates are driving overall rates regardless of age. During World War I, the sex difference in tuberculosis death rates peaked (see Fig 9). The heightened differential is most likely a result of gendered roles as opposed to biological differences since female tuberculosis rates again surpassed male rates from 1924 to 1928, and in 1943 during World War II.

### Respiratory tuberculosis and settlement type

**Fig 10:**
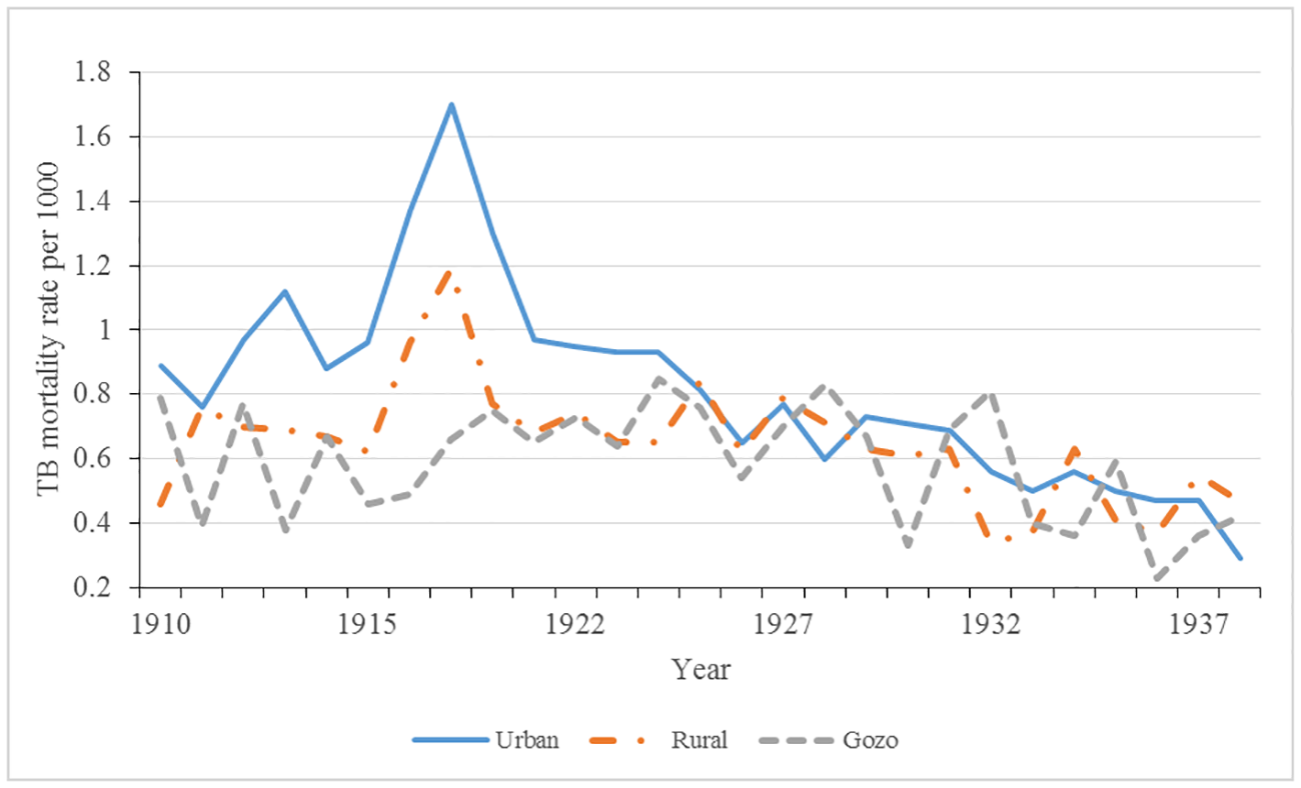
Tuberculosis mortality rates by settlement type: Malta 1910–1938.

Fig 10 depicts the secular trend of respiratory tuberculosis mortality rates by settlement type (Urban, Rural, and Gozo) from 1910 to 1938. The vast regional variations in death rates reveal the importance of hidden heterogeneity of tuberculosis. Urban and rural tuberculosis death rates follow similar trajectories inasmuch as these settlements showed a sharp rise in rates beginning in 1917, peaking in 1918 and returning to pre-1917 rates after the 1920s. Unlike the other two settlement types, Gozo’s tuberculosis death rates did not peak during World War I and had markedly lower tuberculosis rates relative to Malta until the mid-1920s.

### Respiratory tuberculosis and economics

The regression and correlation results shown in Table 1 indicate that respiratory tuberculosis in both urban and rural settlements (in Malta proper) were significantly influenced by the price and inflation of imported food (p<0.0001). For both urban and rural settlements, just over 60% of the variation in tuberculosis death rates can be explained by the cost of living. In Gozo, there was no significant impact on respiratory tuberculosis even as the cost of living rose during the years 1917 to 1919. An examination of overall tuberculosis rates for reproductively aged individuals before and during the war shows a very strong relationship between high tuberculosis death rates and the poor economy. Obviously, the war was a driving force in lower economic levels, and economics explains the general downward trend in tuberculosis rates over the study period.

**Table 1:** Relationship between regional tuberculosis rates and Fisher Index.

	R	p-value
**Region**		
Urban/Suburban	0.782	<0.0001
Rural	0.778	<0.0001
Gozo	0.229	0.25
**Time Period**		
Total (1912–1918)	0.969	<0.001
Total (1919–1938)	0.889	<0.0001

## Discussion

This paper examined the complexity within the secular trend in respiratory tuberculosis in the Maltese islands, noting both the slow progressive decline since the early 20^th^ century as well the sharp increase observed in 1917 and 1918. Undoubtedly, there is considerable complexity at the root of the “causes” of the observed pattern. Nevertheless, we argue that the most parsimonious explanation for the decline and temporary increase can be attributed to changes in the economy and its trickle-down effects. While this explanation was originally proposed by McKeown and colleagues [10] nearly half a century ago, the Malta case study provides evidence of the relationship between a nation state’s economy and tuberculosis rates. Malnutrition is a direct proximal cause for tuberculosis susceptibility and death resulting from the increase in cost of living [12, 38]. Most likely, the lack of meat consumption deprived individuals of dietary B3 and tryptophan and was responsible for increase of tuberculosis infections [39].

Further confirmation of the role of the standard of living and its impact on health can be seen in the results of our regional analysis. Our results fall in line with those of Spain where there is evidence that urban centers with a large concentration of the working poor and abysmal living conditions provided ideal environments for epidemics of respiratory infections and, in particular, respiratory tuberculosis. The “urban penalty” suggests that urban locales such as towns and cities “concentrate poor people and expose them to unhealthy physical and social environments” [40] (pg. 1). Similarly, there were lower rates of tuberculosis deaths in rural areas than urban rates [41]. It has been postulated that rurality protected individuals from mortality during the influenza pandemic in New Zealand because social distancing (lower person-person contact) and remoteness lowered transmission of infection from urban to rural areas [42]. Our findings on Gozo’s low tuberculosis mortality, even in the face of disruption related to World War I, point to the importance of lower exposure owing to a number of conditions: isolation and healthy outdoor agrarian life style; economic self-sufficiency (in addition to being farmers and fishermen, the other chief occupations for men were furniture craftsmen and masons); and relatively low reliance on imported food compared to Malta [23].

As stated earlier, a number of reasons can account for the increase of tuberculosis rates during World War I, but our primary explanation lies in an economic upheaval. As a British colony situated in the heart of the Mediterranean, the largest island of Malta was a strategic stronghold for the British military. During World War I, Malta, a supply station for the British military, was heavily involved in caring for the sick and injured and became known as “the Nurse of the Mediterranean.” Not surprisingly then, the economy of Malta during the course of the war was dependent on providing services for the Royal Navy [43]. As the war progressed, there was a downturn in the war related activities, which culminated in a large number of unemployed men and women. While the war effort initially brought economic prosperity to the Maltese, this prosperity was short lived. By 1916, the cost of the living by doubled; unemployment rates, employment instability, and food shortages rose [44, 45]. In addition, the cost of food items rose; they were inferior in quality and difficult to purchase, even at inflated prices [46]. For example, from 1914 to 1918, high protein food items (fish, meat, cheese, eggs) increased from 200% to 500% [47]. An important food staple was bread as it was the major source of energy especially for the poor. During World War I, the price of wheat increased and the availability of flour decreased significantly, to the point where the price of bread trebled by the end of the war. The shortage of bread was so acute that it contributed to social unrest and a strike at the Malta Dockyard in May of 1917 [44, 45].

Other evidence of the dire state of the economy was the lack of available and new housing, a shortage that certainly would have contributed to overcrowding as large family size in Malta continued unabated. We argue that household security was compromised, not only because of growing rates of unemployment and unstable employment, but also because construction of new housing dwindled during the war years exacerbating the existing state of overcrowding. Stagnation of housing was evident in 1915 when the number of houses built declined from 152 in Malta and 25 in Gozo, to 17 and 5 by 1920–21. This was precisely the period when respiratory rates rose. Only in 1922 was there an improvement in living conditions when a total of 5311 houses in Malta and 370 in Gozo were built from 1922 to 1933 [48]. Not only did overcrowding, unemployment and high prices of food and other necessities fuel a compromise to general health, but the absence of public welfare for the needy further affected the well-being of the Maltese working poor [43].

### Influenza and tuberculosis

Our emphasis on the war and economy stands in opposition to the work of Noymer [49] who singles out the influenza pandemic as a defining moment in the history of tuberculosis and precipitating its decline, especially in males (see also Noymer and Garenne [50]). Furthermore, Noymer [20] postulates that there was a selection effect, specifically passive selective, which resulted in increased tuberculosis mortality during the 1918 pandemic because of the “age-mortality overlap” with influenza (p. 1601). Because of the exceptionally high rates of tuberculosis during World War I, there was a rapid two year decline of tuberculosis death rates after 1918 in the USA [20]. Undoubtedly, the influenza pandemic played a contributing role to the increase in tuberculosis deaths in our study period, but it cannot be the primary factor simply because the rise began a year before the start of the pandemic in September, 1918. Collectively, the augmented tuberculosis rates in 1917 and 1918/1919 in Malta resulted in a rapid decline of tuberculosis rates post-1919 and returned to almost pre-war levels in the overall rates and for females. The decrease in death rates following a stressor period is known as the “harvesting effect” or “short-term mortality displacement.” This phenomenon occurs when there is a heightened, albeit temporary, mortality rate among those with health complications because of underlying health problems (especially cardio-respiratory diseases) and among the elderly [51–52] or because of increased vulnerability associated with lower socio-economic status. Following the spike in deaths, there is a drop in the death rate, the aftermath of the harvesting of the frail segment of a population [53]. The war together with influenza cases accelerated deaths in the tuberculous, who might have otherwise had many more years to live and, very probably, hastened the transition from latent state tuberculosis to full blown tuberculosis since almost all young adults were exposed to the bacillus during this time [9].

The lack of the harvesting effect in males is most likely a result of lower levels of tuberculosis rates. In addition, we must remember that identifying specific events associated with harvesting is fraught with many complications: there is no established method for assessing the harvesting effect. First, the stressor or event cannot be constant in the population, as in the occurrence of extremely hot weather, drought, or pollution [54]. Second, there is no specific time frame defining when the “dip” or healthy period begins and how long it should last [55]. Obviously, the scale of the stressor will determine the length of the healthy period, be it days, weeks, months or years. It is incumbent on the researcher to clarify the extent of the stressor or event and the expected limits of the health period. Fourth, we emphasize one additional requisite condition that determines whether the harvesting effect is operating: there must be an eventual return to background levels; that is, rates should return to pre-stressor levels. Otherwise, harvesting will persist indefinitely or fail to exist. One such example of the misuse of harvesting in this context is the study by Oei and Nishiura (2012) [56] who stated that harvesting of tuberculosis occurred following the influenza epidemic in Japan and the Netherlands. However, because tuberculosis has been declining over time, the return to “normalcy” did not obtain the same level as pre-war rates. Lastly, it is paramount to recognize that harvesting is population specific: it is contingent on the population at risk, age distribution, and causes of death [57].

## Tuberculosis and gender differences

Hudelson [58] suggests that there are a number of factors that have potential implications for gender differentials in tuberculosis morbidity and mortality. Of these, two factors merit consideration for our study. Both center around gendered vulnerabilities: (1) differential exposure to the tuberculosis bacilli; and (2) general health/nutritional status of TB-infected persons.

Our research supports the earlier suggestion that gendered vulnerabilities lie at the root of heightened female respiratory mortality rates that began in 1917. We posit that, as the primary caregivers for the sick within the traditional patriarchal large extended family unit, Maltese women were uniquely placed to be exposed to the bacilli during periods of deprivation and instability. Owing to their gendered role as caregivers, homemakers and to the burden of domestic duties, women underwent markedly higher stress levels as they tried to maintain daily essential elements of household security [59]. Crowded into unsanitary and poor ventilated living quarters, large families provided ideal conditions for spread of infectious diseases spread through continuous contact and close physical and social proximity to one another. Furthermore, the selflessness of the women placed family and husband first, especially when there were food shortages and/or a lack of quality foodstuffs.

Our results on sex differences in tuberculosis rates agree with the findings of Cobbett [37], who, nearly a century ago, reported that females, rather than males, were most affected during the war. From the vantage point of working shortly after World War I, Cobbett concluded that it was not an increase in new cases, but those who were already affected that succumbed to tuberculosis. The rise in mortality came about, he suggested, because nutrition was seriously impaired by the war. In Malta during World War I, the sex differential in adult (aged 15-44years) tuberculosis rates peaked; the heightened differential was a result of increased stressors placed on women to maintain a household when resources were scarce. Obviously, increased tuberculosis rates during the World War I placed a burden on the reproductive fitness of women. During World War II, women bore the brunt of many pressures, and the importance of gendered roles was again thrown into prominence. We cannot ignore that periodically male tuberculosis mortality rates surpassed those of females. Explanations for the change in the sex differential in tuberculosis, which resulted from the transient increase in male rates and the continuation in the decline in female rates, will be explored in future studies.

## Conclusion

Today, tuberculosis along with HIV is one of the leading causes of death [60]. The drain of tuberculosis morbidity and mortality on public health is apparent in both developing and developed nations alike. In developing nations, tuberculosis account for about 26% of avoidable deaths [61] and, in all parts of the world, it is a remerging opportunistic disease in those with HIV and other vulnerable groups. Understanding trends in tuberculosis mortality in past contexts during periods of stability and moments of heightened stress offers insight into disease management when future epidemics occur.

We have reaffirmed that economics, the cost of living in particular, was a major factor in determining tuberculosis mortality rates in the 20^th^ century. Furthermore, we observed the harvesting of deaths of the tuberculous was observed during times of economic strain. The importance of the heterogeneity of regional rates of tuberculosis because of variation in economic dependency within a nation state was demonstrated: Gozo’s experience with tuberculosis was muted primarily because of isolation and a self-sufficient economy.

## Supporting information

S1 Dataset Annual TB deaths and population at risk information for Malta. (XLSX)
